# Age‐associated sex and asymmetry differentiation in hemispheric and lobar cortical ribbon complexity across adulthood: A UK Biobank imaging study

**DOI:** 10.1002/hbm.26076

**Published:** 2022-09-15

**Authors:** Nafeesa Nazlee, Gordon D. Waiter, Anca‐Larisa Sandu

**Affiliations:** ^1^ Aberdeen Biomedical Imaging Centre University of Aberdeen Aberdeen Scotland

**Keywords:** ageing, cortex, cortical complexity, fractal dimension, hemispheric asymmetry, MRI, sexual dimorphism

## Abstract

Cortical morphology changes with ageing and age‐related neurodegenerative diseases. Previous studies suggest that the age effect is more pronounced in the frontal lobe. However, our knowledge of structural complexity changes in male and female brains is still limited. We measured cortical ribbon complexity through fractal dimension (FD) analysis at the hemisphere and lobe level in 7010 individuals from the UK Biobank imaging cohort to study age‐related sex differences (3332 males, age ranged 45–79 years). FD decreases significantly with age and sexual dimorphism exists. With correction for brain size, females showed higher complexity in the left hemisphere and left and right parietal lobes whereas males showed higher complexity in the right temporal and left and right occipital lobes. A nonlinear age effect was observed in the left and right frontal, and right temporal lobes. Differential patterns of age effects were observed in both sexes with relatively more age‐affected regions in males. Significantly higher rightward asymmetries at hemisphere, frontal, parietal, and occipital lobe level and higher leftward asymmetry in temporal lobe were observed. There was no age‐by‐sex‐by asymmetry interaction in any region. When controlling for brain size, the leftward hemispheric, and temporal lobe asymmetry decreased with age. Males had significantly lower asymmetry between hemispheres and higher asymmetry in the parietal and occipital lobes than females. This work provides distinct patterns of age‐related sex and asymmetry differences that can aid in the future development of sex‐specific models of the normal brain to ascribe cognitive functional significance of these patterns in ageing.

## Graphical abstract

Brain cortical complexity, measured through fractal dimension analysis, decreases with increasing age and parietal lobe is the most age affected lobe in both sexes across adulthood (age range 45–79 years). Males, having significantly higher cortical complexity than females, showed greater complexity decline in right hemisphere. Markedly different age and asymmetry patterns were noted in both sexes. These findings can help in future development of sex‐specific models of normal brain to ascribe cognitive functional significance of these patterns in ageing.

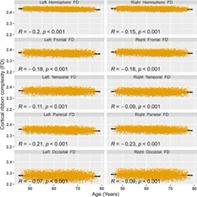

## 1 INTRODUCTION

The human brain has a complex shape, and its stereotyped pattern of gyri and sulci allows an increase in grey matter (GM) volume. Studying regional changes in GM complexity in the normal brain across adulthood is important for understanding human brain function, clinical diagnosis, and distinguishing between normal ageing and neurological disorders whose risk increases with advancing age (e.g., dementia and Alzheimer's disease). Fractal dimension (FD), which describes cortical shape complexity within the folds and ridges of neuroanatomical regions is a useful biomarker of ageing. FD of the cortical ribbon is found to be highly correlated with chronological age and dementia diagnosis (King et al., 2010; Madan & Kensinger, 2016), and can also detect the subtle changes present in the early stages of diseases, for example, in multiple sclerosis (Esteban et al., 2009). Age has a pronounced and differential effect on regional cortical complexity (Liu et al., 2020; Lu, 2020; Madan & Kensinger, 2016), yet, little is known about sexual dimorphism in age‐related regional cortical complexity decline across adulthood.

It is well known that age‐related cortical atrophy occurs heterogeneously across the cerebral cortex and MRI studies that have investigated age‐related differences using measures such as regional brain volumes, cortical thickness, and GM density have found that the most age‐affected lobes are the frontal and temporal lobes, while the occipital lobe is least affected (Allen et al., 2005; Bourisly et al., 2015; Coffey et al., 1992; Fjell et al., 2009; Fjell & Walhovd, 2010; Raz et al., 2004; Sowell et al., 2003). Using the Destrieux parcellation scheme, Madan and Kensinger (2016) measured shape‐related characteristics with FD in bilateral structures of each lobe (*n* = 581, age ranged 20–86) and reported that age‐related decrease in FD was highest in the frontal lobe followed by the parietal lobe while the temporal lobe was least associated with age‐related difference. Recently, Jao et al. (2021) studied subregional cortical changes using 3D‐FD analysis with Desikan–– (DK) atlas (*n* = 258, age ranged 30–85 years) and reported a decrease in cortical complexity of all lobes of both hemispheres with age progression from adulthood to old age. The temporal, parietal, and left limbic lobes exhibited a significant decrease from mid‐life (45–60 years) to old age (>60 years). The reduction in FD was more noticeable in bilateral rostral middle frontal, middle temporal, superior temporal, transverse temporal, superior parietal, and lateral occipital regions in old age. Moreover, an increase in FD was observed in the occipital lobe in old age (Jao et al., 2021). These findings suggest that age‐related changes in FD are not uniform across different stages of ageing.

It has been shown that FD represents an extremely compact measure of shape complexity, condensing cortical thickness, sulcal depth, and folding area into a single numerical value—all these indexes being, in fact, closely linked (Im et al., 2006). Earlier studies employing this method demonstrated that FD is more sensitive to age‐related cortical atrophy than other surface‐based measurements like cortical thickness or gyrification index (Lu, 2020; Madan & Kensinger, 2016; Madan & Kensinger, 2017b; Madan & Kensinger, 2018). More importantly, several studies have found that information conveyed by FD is additional to that provided by other conventional structural measures (Free et al., 1996; King et al., 2009; King et al., 2010; Lu, 2020; Madan & Kensinger, 2016; Madan & Kensinger, 2018). FD can detect changes in the structural complexity of the cerebral cortex in healthy ageing (Krohn et al., 2019; Liu et al., 2020; Madan & Kensinger, 2016; Marzi et al., 2020; Sandu, Staff, et al., 2014), neurological disorders (King et al., 2016; Ruiz de Miras et al., 2017; Sandu, Martinot, et al., 2014; Sheelakumari et al., 2018) and neuropsychological disorders (Narr et al., 2004; Nenadic et al., 2014; Nickel et al., 2019; Sandu, Rasmussen Jr., et al., 2008; Sandu, Specht, et al., 2008). Normal brain morphological alterations associated with ageing do not occur homogeneously and are region‐specific, thus region‐specific FD analysis might serve as a biomarker to track therapeutic interventions targeted at disorders associated with accelerated atrophy such as dementia and disease progression such as spinocerebellar ataxia (Marzi et al., 2018).

Sex is recognized to be an important biological variable in brain research (Xin et al., 2019), and many prior MRI studies observed sex differences in brain volumes—on average, males have larger brain GM volumes than females (Allen et al., 2003; Giorgio et al., 2010; Good et al., 2001; Lemaitre et al., 2012; Smith et al., 2007; Terribilli et al., 2011) while females have a thicker cortex (Herron et al., 2015; Sowell et al., 2007); however, no sex differences are reported in gyrification (Hogstrom et al., 2013). It is important to note that the reported sex differences in studies that do not account for brain size differ considerably from those that do perform brain size correction (Luders & Kurth, 2020). Studies have suggested that after controlling for the effect of total percent brain volume change, men showed greater relative regional brain reduction than women in bilateral precentral gyri, bilateral paracingulate gyri, and bilateral supplementary motor cortices in mid‐life (Guo et al., 2016). Recently, Jao et al. (2021) reported the effects of normal ageing on regional cortex complexity in men and women (112 women, 146 men). They reported that middle‐aged men exhibited an earlier and more significant decrease in FD values mainly in the right frontal and left parietal lobes, than did middle‐aged women and the sex differences during normal ageing continued as age progressed with more morphological alterations in men than in women. However, the above reported age and sex differences were not corrected for brain size.

Left–right hemispheric asymmetry is a key feature of human brain structure and is considered to be partly responsible for functional lateralization including language and behaviour. For example, some language‐related regions showed greater average left than right surface areas, including superior temporal and supramarginal cortex and pars opercularis (Sha et al., 2021). Lee et al. (2004) measured the GM hemispheric skeleton asymmetry in 62 participants with FD and showed significant rightward asymmetry that did not change according to age and gender. A recent study suggests that during young adulthood, women exhibited more cortical lateralization than men (Jao et al., 2021). With progression to middle age, men exhibited increased cortical lateralization with 24 subregions having significant asymmetrical differences where smaller FD values in the left hemisphere were found in frontal, temporal, and parietal lobes (left/right = 15/9).

In the present study, we examined the effects of normal ageing on the hemisphere and regional (unilateral frontal, temporal, parietal, and occipital lobes) cortical structures across adulthood (age range 45–79 years) in a large population cohort providing a unique description of age‐related change and sexual dimorphism in brain complexity. We investigated the age, sex, and asymmetry differences in cortical ribbon complexity quantified through FD in middle to older age adults. Tracing the patterns of age‐related changes in cortical complexity may provide a suitable tool for the assessment of normal ageing and neurodegeneration between groups or in individuals at high risk of developing cognitive impairment in later stages of normal ageing.

## 2 MATERIALS AND METHODS

### 2.1 Participants

UK Biobank (http://www.ukbiobank.ac.uk/) is a large, population‐based biomedical study. Between 2006 and 2010, around 500,000 participants were recruited from across Great Britain and attended one of 22 assessment centres where they underwent extensive behavioural and cognitive assessment and the collection of demographic and lifestyle information (Allen et al., 2012). A subset of these participants also had a brain MRI scan at a single centre between 2014 and 2016 (Miller et al., 2016). From the January 2017 brain imaging data release, 7375 participants aged between 45 and 79 years (mean = 62.5, SD = 7.4 years) without any neurological or psychiatric condition were included in this cross‐sectional study. There were 3677 females (mean age = 61.75, SD = 7.23) and 3332 males (mean age = 63.28, SD = 7.47). All UK Biobank participants gave written informed consent. UK Biobank received ethical approval from the Northwest Multi‐Centre Research Ethics Committee (11/NW/03820). This research was conducted using the UK Biobank Resource under Application Number 24089 (PI Waiter). All UK Biobank methods were performed following the UK regulations (https://www.ukbiobank.ac.uk/gdpr/).

### 2.2 Brain image acquisition and processing

MRI data for all participants were acquired on a single Siemens Skyra 3 T scanner, according to previously reported procedures (Miller et al., 2016). These data included a 3D MPRAGE T1‐weighted MRI scan which were pre‐processed using a developmental version of FreeSurfer v 6.0 software package (http://surfer.nmr.mgh.harvard.edu). FreeSurfer provides full processing streams for volume‐based and surface‐based structural MRI data (Fischl et al., 2001; Fischl & Dale, 2000). The pre‐processing steps include skull stripping, B1 bias field correction, and GM–white matter (WM) segmentation followed by initial volumetric labelling. In surface‐based processing, it reconstructs cortical surface models by defining the boundary between WM and cortical GM to determine the WM surface (white–grey interface) and the pial surface (grey‐cerebrospinal fluid interface) and does labelling of regions on the cortical surface. These surfaces were automatically corrected for topological defects (Fischl et al., 2001; Ségonne et al., 2005) and no manual edits were made to these surface models (e.g., manual trimming). Since the quality control of Freesurfer outputs is of paramount importance, the subjects in this study were the same who passed the quality control proposed by Alfaro‐Almagro et al. (2018). FreeSurfer also provides the cortical ribbon which is unparcellated GM and segmented left and right hemisphere cortical ribbons as intermediate output files during the analyses. For cortical ribbon GM parcellation in the left and right hemispheres corresponding to each lobe, we used gyral‐based parcellation protocol (Desikan et al., 2006) where each of the 34 parcellated regions was dummy‐coded by lobe. An in‐house Statistical Parametric Mapping (SPM12) toolbox which ran on MATLAB (R2018a, The MathWorks Inc., Natick, MA) was designed to group parcellated regions of interest (ROIs) that assigned the same dummy‐coded labels (1000 for left and 2000 for right) into binarized volume, before calculating the FDs of unilateral lobes. The details of the ROIs with their respective ROIs locations in each lobe are described in Table 1.

**TABLE 1:** ROIs in DK atlas

Frontal	ROI	Temporal	ROI
1003	Left caudal middle frontal	1001	Left Bankssts
1012	Left lateral orbitofrontal	1006	Left entorhinal
1014	Left medial orbitofrontal	1007	Left fusiform
1017	Left paracentral	1009	Left inferior temporal
1018	Left pars opercularis	1015	Left middle temporal
1019	Left pars orbitalis	1016	Left parahippocampal
1020	Left pars triangularis	1030	Left superior temporal
1024	Left precentral	1033	Left temporal pole
1027	Left rostral middle frontal	1034	Left transverse temporal
1028	Left superior frontal	*Parietal*	
1032	Left frontal pole	1008	Left inferior parietal
Occipital		1022	Left postcentral
1005	Left cuneus	1025	Left precuneus
1011	Left lateral occipital	1029	Left superior parietal
1013	Left lingual	1031	Left supramarginal
1021	Left pericalcarine		

Abbreviations: DK, Desikan–Killiany; ROI, regions of interest.

### 2.3 Three‐dimensional FD

To characterize the complexity of the left and right hemisphere cortical ribbon, the mask of cortical GM, (i.e., *lh.ribbon.mgz* and *rh.ribbon.mgz*), created during FreeSurfer pre‐processing, served as the basis for the estimation of FD. Among several approaches available to calculate FD, the box‐counting method is more widely used due to its robustness and is considered more reliable to analyse fractal objects, the brain being a natural fractal (Farahibozorg et al., 2015; Madan & Kensinger, 2016; Mustafa et al., 2012; Pantoni et al., 2019; Sandu, Izard, et al., 2014). We used an FD toolbox in MATLAB 2018a based on a box‐counting algorithm which was developed and validated by Sandu, Rasmussen Jr., et al. (2008) and has been used previously (Sandu, Izard, et al., 2014; Sandu, Staff, et al., 2014) and recently (Sandu et al., 2022). Briefly, a fixed grid composed of 3D boxes (i.e., voxels) of edge length (*r*) is overlapped to the brain ROI (parcellated cortical GM), and the number of grid boxes *N*(*r*) containing at least one voxel belonging to cortical ribbon is counted. The edge length of boxes (*r*) is increased linearly and the number of filled boxes *N*(*r*) is counted again each time. This relationship follows a power law *r* ~ *N*(*r*)^−FD^ and is determined by plotting box edge length (*r*) versus related box counts *N*(*r*) on a double log plot. The absolute value of the slope provides the FD value of the structure. We increased the edge length of boxes as one voxel per iteration, within the range from *r* = 2 to *r* = 30 voxels for hemisphere cortical ribbon and *r* = 2 to *r* = 14 voxels for lobes cortical ribbon images.

### 2.4 Statistical analysis

To track the patterns of age‐associated change in complexity, we regressed the age over each FD measure using simple linear regression models for all participants and each sex individually. We performed the multiple linear regressions including age and sex as main effects, and total brain volume (TBV) as confounding factors. Since each FD measure was positively correlated with TBV (*p* < .001), therefore TBV was included as covariate in the model. To evaluate the nonlinear effect of age on each FD, we included the age^2^ term in the second set of analyses and compared the fit of both models with the Akaike information criteria (AIC). The lower the AIC, the better the model fit, that is, ∆AIC between the two models is <2 (Burnham & Anderson, 2002). We analysed the effects of age on each FD measure in males and females separately. Further, we categorized the data set into smaller age subgroups with 5 years age difference. The age effects on each age subgroups were assessed with multiple linear regressions including age as main effect and sex and TBV as confounders.

To examine whether cortical complexity measures were more strongly associated with age in males or females, we repeated the multiple linear regressions, with FD as the dependent variable and age × sex as independent variables and TBV as a confounder.

We computed the normalized hemispheric asymmetry (2 × (left − right)/(left + right)) for hemisphere and each lobe FD measure. To evaluate the main effect of asymmetry, we adopted a systematic statistical approach of using multiple linear regression models with three‐way interactions to account for enough of the variance that the main effect could account for and whether it depends on the other independent variable (age and sex) or not. Direction of asymmetry was determined with paired sample *t* test. Moreover, the main effect of age and sex on asymmetry measures was determined through multiple linear regression with TBV as confounder.

Outliers in the FD measurement were checked using *identify_outliers* function in R for each FD measurement separately, 365 data points were identified as extreme outliers which were removed before analysis. The data was approximately normally distributed as checked by QQ‐plots and variances were homogeneous, as assessed by Levene's test (*p* > .005). The *p*‐values reported here are corrected for multiple comparisons to minimize the spurious effect of type I statistical errors (Armstrong, 2014). The alpha significance levels were set at *p* (.05/10) = .005 for estimated effects of age sex and asymmetry multiple linear regressions and *p* (.05/5) = .01 for age and sex effects on asymmetry measures.

## 3 RESULTS

### 3.1 Demographic characteristics

We measured the structural complexity of the individual hemispheres and lobes in a total of 7010 middle‐aged to older participants aged between 45 and 79 years (mean age 62.48 ± 7.4 years). The cohort included 52.46% females with males being significantly older than females. In our participants, most of them were right‐handed (89%) and a small proportion of participants (2%) were those who used both hands equally for writing. Mean complexity values for hemispheres and lobes for all participants and each sex are reported in Table 2. The total study population was divided into seven age subgroups to map complexity decline trajectories: (i) 45–50 years, mean age = 48.68 ± 0.92 years, *n* = 394, 221 females; (ii) 50–55 years, mean age = 52.55 ± 1.41 years, *n* = 983, 569 females; (iii) 55–60 years, mean age = 57.59 ± 1.44 years, *n* = 1162, 685 females, (iv) 60–65 years, mean age = 62.56 ± 1.42 years, *n* = 1547, 852 females; (v) 65–70 years, mean age = 67.47 ± 1.38 years, *n* = 1771, 864 females; (vi) 70–75 years, mean age = 72.13 ± 1.39 years, *n* = 958, 417 females; and (vi) 75–80 years, mean age = 76.09 ± 0.76 years, *n* = 194, 70 females. In age subgroups, males were older than females only in the youngest age group (age range 45–50 years, *p* = .026) and no effect of sex on age was seen in other groups (*p* > .05).

**TABLE 2:** Basic statistics of cortical ribbon complexity measured through FD (mean, SD) in the UK Biobank data set for all participants and females and males and Welch *t*‐test statistics (*t*, *p* value) for sex group comparison

	All participants (*n* = 7010)		Female (*n* = 3678)		Male (*n* = 3332)		*t*‐Test statistics	
	Mean	SD	Mean	SD	Mean	SD	*t*	*p*‐Value
Age	62.47	7.39	61.75	7.23	63.28	7.47	−8.67	<2.2e‐16
*Hemisphere*								
Left	2.428187	0.0106	2.42639	0.0102	2.43016	0.0106	−15.128	<2.2e‐16
Right	2.429761	0.0112	2.42713	0.0109	2.43265	0.0108	−21.277	<2.2e‐16
*Frontal lobe*								
Left	2.367612	0.0151	2.36576	0.0150	2.36965	0.0150	−10.827	<2.2e‐16
Right	2.373033	0.0147	2.37155	0.0146	2.37467	0.0147	−8.8715	<2.2e‐16
*Temporal lobe*								
Left	2.36332	0.0164	2.36076	0.0164	2.36614	0.0159	−13.94	<2.2e‐16
Right	2.356439	0.0163	2.35264	0.0157	2.36064	0.0159	−21.141	<2.2e‐16
*Parietal lobe*								
Left	2.349877	0.0163	2.35011	0.0165	2.34962	0.0161	1.3128	0.1893
Right	2.35355	0.0160	2.35293	0.0160	2.35425	0.0160	−3.5127	0.0004
*Occipital lobe*								
Left	2.273693	0.0227	2.27107	0.0228	2.27655	0.0222	−10.087	<2.2e‐16
Right	2.283624	0.0226	2.28008	0.0220	2.28755	0.0226	−13.896	<2.2e‐16
Total brain volume	1,102,878.0	101,567.20	1,051,176.0	79,037.94	1,159,948.0	92,741.84	−52.578	<2.2e‐16

*Note*: FD is a unitless quantity and measured through the box‐counting method, total brain volume (cm^3^) is estimated with Freesurfer 6.0 version.

Abbreviation: FD, fractal dimension.

The results of the evaluation of age, sex, and asymmetry differences in FD are reported in the following paragraphs.

### 3.2 Significant effect of age on brain regional cortical ribbon complexity

**FIGURE 1:**
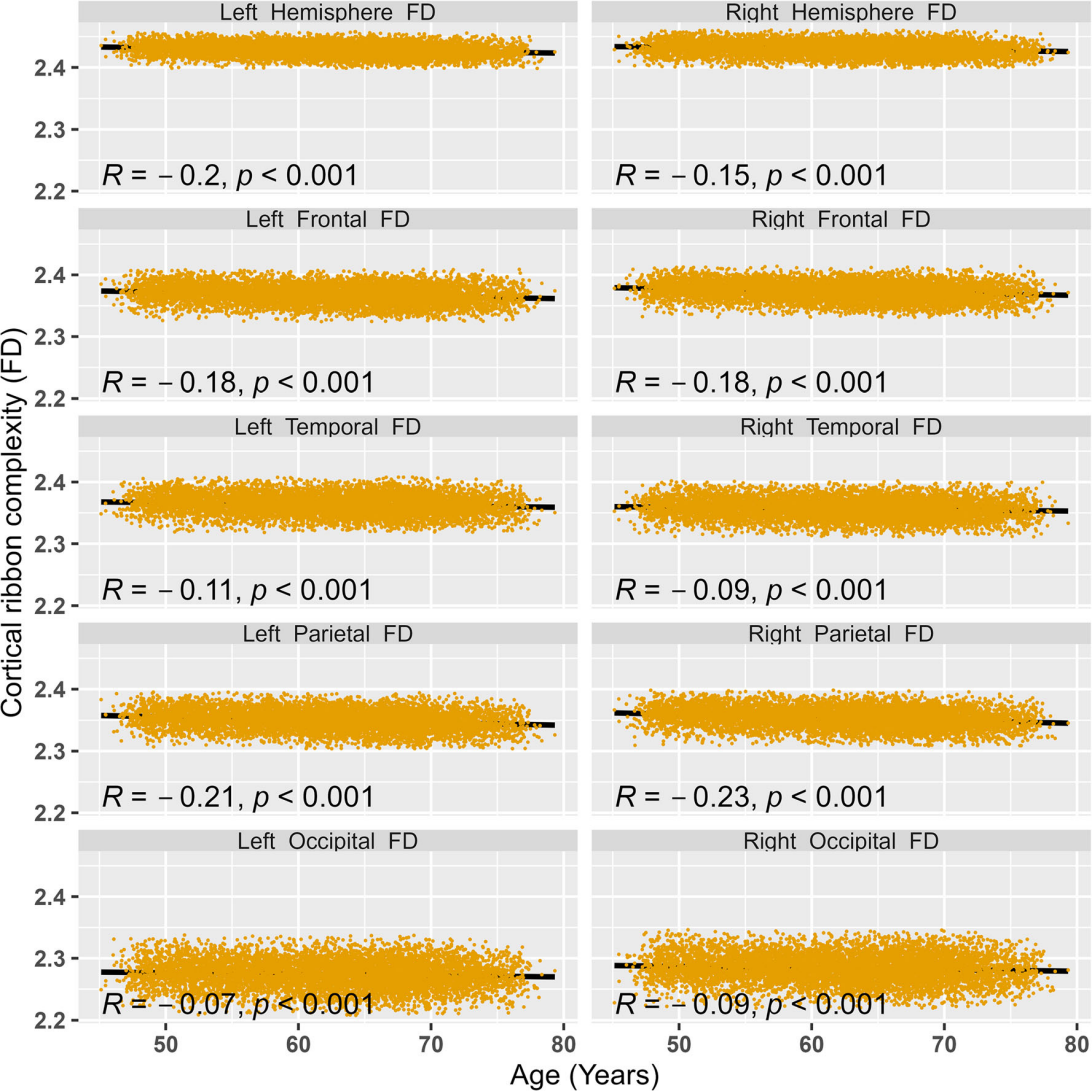
Linear relationship between cortical ribbon complexity (fractal dimension [FD]) and age for all participants (*n* = 7010)

Higher FD values represent a more complex structure. We used the unilateral structure of the hemisphere and each lobe in our analysis. Globally, the right hemisphere and regionally, the right frontal lobe were among the most complex structures in both males and females (Table 2). Overall, the right brain was found to be more complex than the left brain in both sex groups. To track the patterns of age‐associated change in complexity, we regressed the age over each FD measure using simple linear regression models (Figure 1). The FD significantly decreases with age in all regions and the age effect was more dominant at the left hemisphere and right parietal lobe. The pattern of age effect remained consistent even after adjusting for brain size (TBV) and sex (Table 3). The nonlinear effect of age was significant at left and right frontal lobe and right temporal lobe, that is, lower AIC for age^2^ model in comparison to only age effect model (Table 3).

**TABLE 3:** Estimated effects of age, sex, and age^2^ (*b*_o_ [intercept], estimates, standard error, and significance *p*‐value) on each FD measure, model fit is measured through AIC

		Age effect			Sex effect				Age^2^ effect			
	*b*_o_	Estimate	SE	*p*‐Value	Estimate	SE	*p*‐Value	AIC	Estimate	SE	*p*‐Value	AIC
*Hemisphere*												
Left	2.38399	−0.0002	1.57E‐05	4.71E‐22	−0.0014	0.0003	4.64E‐07	−45,715.4	2.02E‐06	1.96E‐06	.3026	−45,714.42
Right	2.37428	−0.0001	1.63E‐05	1.80E‐08	−0.0004	0.0003	.1514	−45,216.83	−5.88E‐08	2.03E‐06	.9769	−45,214.83
*Frontal lobe*												
Left	2.34641	−0.0003	2.44E‐05	2.17E‐29	0.0005	0.0004	.2056	−39,521.6	9.60E‐06	3.04E‐06	.0016	−39,529.58
Right	2.35161	−0.0003	2.39E‐05	1.04E‐28	−0.0003	0.0004	.5401	−39,852.1	1.15E‐05	2.97E‐06	.0001	−39,864.98
*Temporal lobe*												
Left	2.32761	−0.0002	2.65E‐05	2.69E‐09	0.0012	0.0005	.0098	−38,373.3	3.62E‐06	3.31E‐06	.2736	−38,372.48
Right	2.29513	−0.0001	2.53E‐05	0.005037	0.0017	0.0004	9.18E‐05	−39,055.1	7.29E‐06	3.15E‐06	.0206	−39,058.49
*Parietal lobe*												
Left	2.30984	−0.0003	2.59E‐05	1.13E‐26	−0.0060	0.0004	4.49E‐41	−38,687.5	5.49E‐06	3.23E‐06	.0895	−38,688.43
Right	2.31595	−0.0003	2.52E‐05	1.84E‐37	−0.0041	0.0004	1.22E‐20	−39,070.8	6.01E‐06	3.14E‐06	.0561	−39,072.48
*Occipital lobe*												
Left	2.24495	−0.0002	3.78E‐05	3.88E‐05	0.0020	0.0006	.0025	−33,428.5	2.17E‐06	4.70E‐06	.6443	−33,426.73
Right	2.25451	−0.0002	3.72E‐05	6.41E‐08	0.0038	0.0006	3.33E‐09	−33,619.7	−7.72E‐08	4.64E‐06	.9867	−33,617.73

*Note*: The significance level is set at *p* = (.05/10) = .005 for multiple comparisons.

Abbreviations: AIC, Akaike information criteria; FD, fractal dimension.

### 3.3 Significant effect of age on male and female brain regional cortical ribbon complexity

**FIGURE 2:**
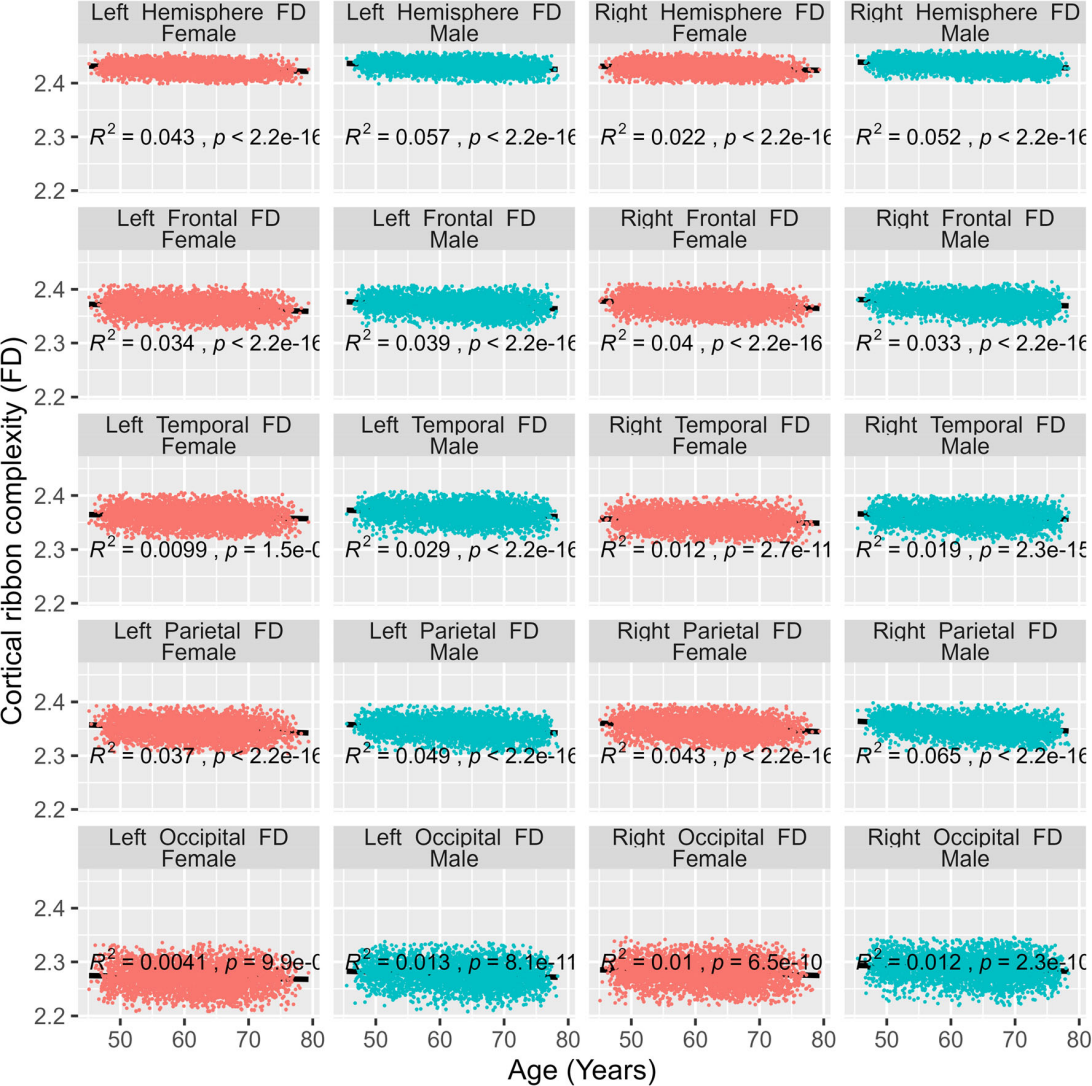
Linear relationship between age and cortical complexity measured through fractal dimension (fractal dimension [FD]) in females and males in left and right hemisphere (top row) followed by frontal (second row), temporal (third row), parietal (fourth row), and occipital (bottom row) lobes. The x axis represents the age at the time of scan (years), and the y axis represents the cortical ribbon complexity measured through FD which is a unitless quantity. *R* ^2^ represents the correlation coefficient and *p* is the significance value, significance level was set at *p* (.05/20) = .0025 after applying Bonferroni correction.

We regressed each complexity measure over age for both males and females separately (Figure 2). The age effects were found to be quite variable across regions and sex groups. We found that the age effect was more pronounced for males (as higher *R* ^2^—correlation coefficient values) except in the right frontal lobe where females showed a slightly higher age effect than in males (females *R* ^2^ = .039 > males *R* ^2^ = .033). At the hemisphere level, the female left hemisphere showed a more pronounced age effect than the right hemisphere which was not true for males. At lobar level, the female right brain was slightly more age affected, the age effect pattern was right parietal > right frontal > left parietal > left frontal > right temporal lobes, and age effects were negligible in left temporal and left and right occipital lobes. In the male, the left brain showed slightly higher age effects than the right side except for the parietal lobe and followed a pattern of right parietal > left parietal > left frontal > right frontal > left temporal > right temporal lobes. The age effects in individual male and female brains with the adjustment of TBV are presented in Table 4. Noticeably, males left temporal and left occipital lobes had a more pronounced age effects in comparison to females. The results from the multiple regression analysis were not much different from the above when the effect of brain size was controlled by including TBV as a confounder. We did not find any age‐by‐sex interaction in any region (*p*‐value > .005) when age × by sex interaction term was included in the model, however, the overall regression models were significant.

**TABLE 4:** Estimated effects of age (*b*_o_ [intercept], estimates, standard error, and significance *p*‐value) on each FD measure in females and males

	Females			Males		
	*b*_o_	Estimate	*p*‐Value	*b*_o_	Estimate	*p*‐Value
*Hemisphere*						
Left	2.3798	−1.46E‐04	1.5E−11	2.38652	‐1.60E‐04	3.93E‐12
Right	2.3579	−3.29E‐05	.1392	2.38991	−1.57E‐04	3.69E‐11
*Frontal lobe*						
Left	2.3446	−2.83E‐04	1.4E‐16	2.34841	−2.70E‐04	1.94E‐14
Right	2.3516	−3.01E‐04	1.7E‐19	2.35067	−2.30E‐04	2.55E‐11
*Temporal lobe*						
Left	2.3114	−8.24E‐05	.0274	2.34496	−2.41E‐04	1.60E‐10
Right	2.2930	−6.91E‐05	.0489	2.29884	−7.33E‐05	0.0446
*Parietal lobe*						
Left	2.2957	−2.54E‐04	2.6E‐12	2.31704	−3.07E‐04	1.88E‐16
Right	2.3108	−2.99E‐04	3.4E‐17	2.31710	−3.53E‐04	2.83E‐22
*Occipital lobe*						
Left	2.2240	−6.75E‐05	.2031	2.26761	−2.52E‐04	2.69E‐06
Right	2.2359	−1.66E‐04	.0011	2.27590	−2.43E‐04	8.78E‐06

*Note*: The significance level is set at *p* = (.05/10) = .005 for multiple comparisons.

Abbreviation: FD, fractal dimension.

### 3.4 Significant effects of sex on brain regional cortical ribbon complexity

Males have significantly higher TBV and higher mean FD values in all regions except the left parietal lobe (Table 2). While accounting for brain size, the sex showed a significant effect in the left hemisphere and left and right parietal lobe where females had significantly higher complexity than males whereas males had higher complexity than females in right temporal and left and right occipital lobes (*p* < .005). Sex effect was not significant in the right hemisphere and left and right frontal, and left temporal lobe (*p* > .005, Table 3). It seems that the sex effect was more pronounced in the left parietal lobe (*p* = 4.49E‐41) where females had significantly higher complexity than males. Our results confirmed that sex differences in brain cortical complexity exist, which provides evidence in favour of the hypothesis that males differ from females on global and regional GM complexities when the issue of overall brain size in the analysis of sex differences was addressed (Rippon et al., 2014).

### 3.5 Significant effects of asymmetry in regional cortical complexity

Paired sample *t* test revealed significantly higher rightward asymmetry (right > left) at hemisphere, frontal, parietal, and occipital lobes whereas the temporal lobe showed the leftward asymmetry (left > right). Results from multiple linear regression models with three‐way interactions are presented in (Table 5). There was no significant age‐by‐sex‐by‐asymmetry interaction, neither age‐by‐sex interaction, nor sex‐by‐asymmetry interaction in any region (*p* > .05). Overall, the main effect of asymmetries was significant in all regions (*p* < .005) though relatively smaller in left hemisphere (unadjusted *p* = .038) and right frontal lobe (unadjusted *p* = .0271).

**TABLE 5:** Estimated effects of asymmetry (estimates, significance *p*‐value) on each FD measure and model fit statistics (adjusted *R* ^2^, significance *p*‐value) including three‐way interaction in the model

	Age		Sex		Asymmetry		Age × sex		Age × asymmetry		Sex × asymmetry		Age × sex × asymmetry		Model fit	
	Estimates	*p*‐Value	Estimates	*p*‐Value	Estimates	*p*‐Value	Estimates	*p*‐Value	Estimates	*p*‐Value	Estimates	*p*‐Value	Estimates	*p*‐Value	Adj. *R* ^2^	*p*‐Value
*Hemisphere*																
Left	−2.66E‐04	<2.2E‐16	−8.80E‐03	1.51E‐05	0.874	0.0386	5.96E‐05	.065	4.01E‐03	.953	−2.54E‐01	.687	7.29E‐03	.464	.1677	<2.2E‐16
Right	−2.66E‐04	<2.2E‐16	8.81E‐03	1.51E‐05	−1.568	2.11E‐04	−5.97E‐05	.065	0.0006	.924	−0.2598	.680	0.0073	.463	.2578	<2.2E‐16
*Frontal lobe*																
Left	−4.10E‐04	<2.2E‐16	2.00E‐03	.487	1.661	4.48E‐07	3.21E‐05	.482	−0.0064	.231	−0.3290	.489	0.0049	.515	.2628	<2.2E‐16
Right	−4.10E‐04	<2.2E‐16	2.01E‐03	.486	−0.729	0.0271	3.20E‐05	.485	−0.0060	.258	−0.3323	.486	0.0049	.516	.2208	<2.2E‐16
*Temporal lobe*																
Left	−2.44E‐04	3.31E‐12	1.30E‐02	.000	1.077	1.50E‐04	−8.72E‐05	.071	0.0033	.471	−3.80E‐02	.926	−0.0014	.828	.3048	<2.2E‐16
Right	−2.44E‐04	2.91E‐12	1.30E‐02	.000	−1.289	5.42E‐06	−8.74E‐05	.069	0.0034	.449	−0.0580	.887	−0.0012	.854	.302	<2.2E‐16
*Parietal lobe*																
Left	−4.42E‐04	<2.2E‐16	5.96E‐03	.046	0.854	0.0069	−7.70E‐05	.104	0.0067	.186	0.5100	.280	−0.0089	.237	.2694	<2.2E‐16
Right	−4.42E‐04	<2.2E‐16	5.95E‐03	.047	−1.522	1.50E‐06	−7.68E‐05	.105	0.0071	.162	0.5063	.284	−0.0088	.240	.2380	<2.2E‐16
*Occipital lobe*																
Left	−2.73E‐04	<2.2E‐16	1.13E‐02	.006	1.451	2.11E‐09	−7.86E‐05	.233	−0.0037	.334	−0.0930	.797	−0.0002	.978	.3209	<2.2E‐16
Right	−2.73E‐04	1.74E‐09	1.14E‐02	.007	−0.841	5.44E‐04	−7.87E‐05	.235	−0.0035	.375	−0.1034	.776	−0.0001	.983	.3091	<2.2E‐16

*Note*: The significance level is set at *p* = (.05/10) = .005 for multiple comparisons.

Abbreviation: FD, fractal dimension.

Mean asymmetry ± SD for males and females are given in Table 6, while controlling the effect of brain size, the main effect of age was significant on leftward hemispheric and temporal lobe (*p* < .001) asymmetries which decrease with age (negative slopes with positive intercepts). The sex effect on asymmetry was significant at the hemisphere, parietal, and occipital lobe (*p* < .01). Our results indicate that the males have significantly lower leftward hemispheric asymmetry, and higher rightward parietal and occipital asymmetry in comparison to females.

**TABLE 6:** Basic statistics of asymmetry (mean, SD) and estimated effects of age and sex (*b*_o_ [intercept], estimates, standard error, and significance *p*‐value) on each asymmetry measure

	Females (*n* = 3678)	Males (*n* = 3332)	Intercept		Age effect on asymmetry			Sex effect on asymmetry		
	Mean SD	Mean SD	*b*_o_	SE	Estimate	SE	*p*‐Value	Estimate	SE	*p*‐Value
Hemisphere	(−0.0003, 0.003)	(−0.0010, 0.003)	3.99E‐03	6.55E‐04	−2.50E‐05	5.32E‐06	2.80E‐06	−3.97E‐04	9.16E‐05	1.48E‐05
Frontal lobe	(−0.0024, 0.006)	(−0.0021, 0.005)	−2.23E‐03	1.15E‐03	−4.32E‐06	9.36E‐06	.6441	3.31E‐04	1.61E‐04	.0401
Temporal lobe	(0.0035, 0.007)	(0.0023, 0.007)	1.38E‐02	1.42E‐03	−3.68E‐05	1.15E‐05	.0014	−2.22E‐04	1.99E‐04	.2631
Parietal lobe	(−0.0012, 0.006)	(−0.0020, 0.006)	−2.63E‐03	1.30E‐03	1.95E‐05	1.05E‐05	.0651	−8.42E‐04	1.82E‐04	3.58E‐06
Occipital lobe	(−0.0040, 0.011)	(−0.0048, 0.011)	−4.29E‐03	2.21E‐03	2.02E‐05	1.80E‐05	.2621	−7.94E‐04	3.10E‐04	.0104

*Note*: The significance level is set at *p* = (.05/5) = .01 for multiple comparisons.

### 3.6 Trajectories of age‐associated change in cortical complexity

**FIGURE 3:**
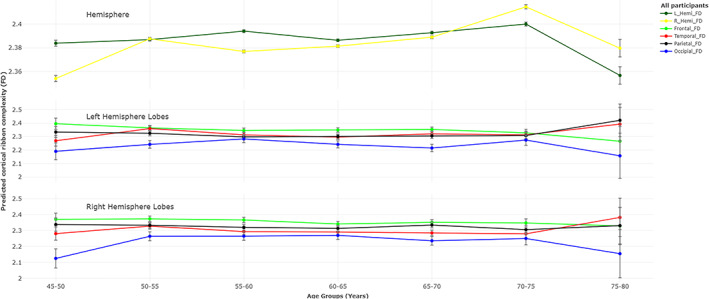
Predicted values of cortical complexity (fractal dimension [FD]) with standard error (SE) of the hemispheres and each lobe for each age subgroup.

**FIGURE 4:**
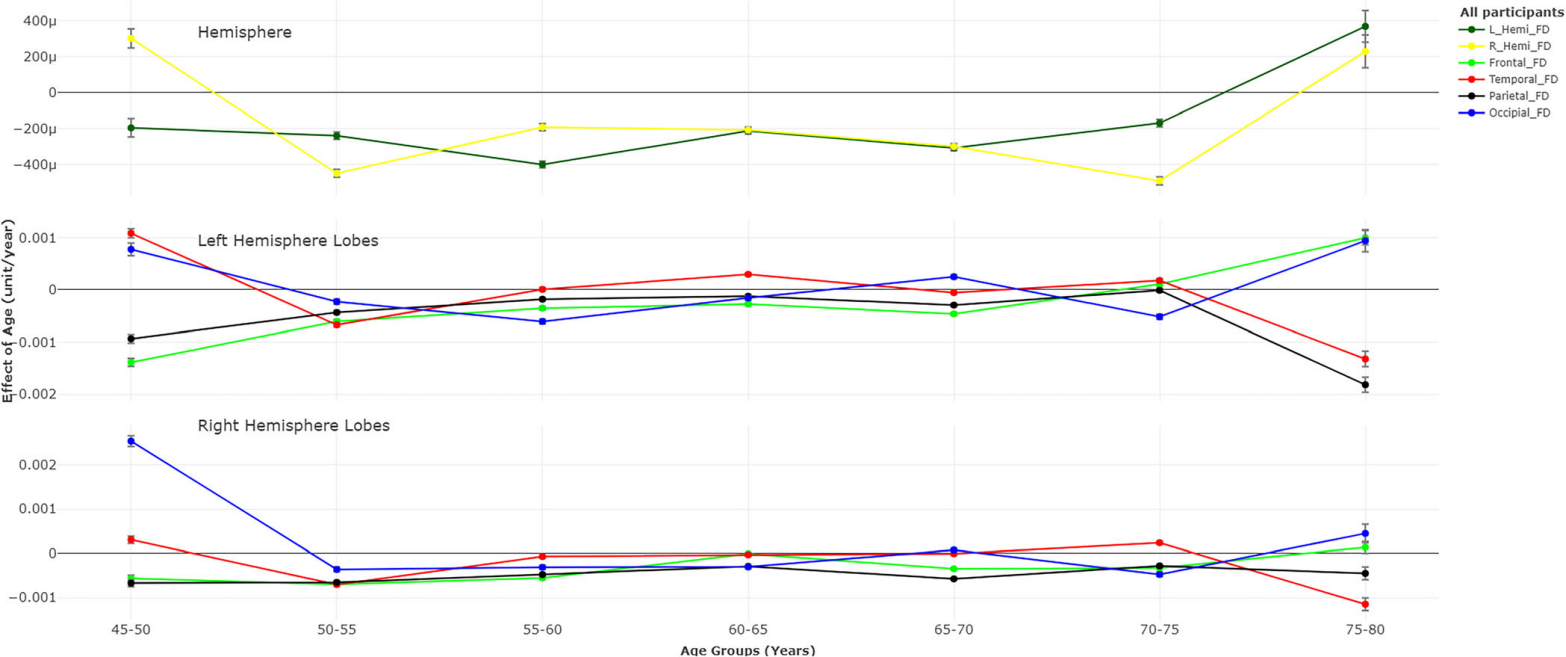
Estimates with standard error (SE) of effects of age on the hemispheres and each lobar fractal dimension (FD) for each age subgroup.

The estimates of effects of age on each FD measure, while controlling for sex and TBV, for each age group are given in Table 7, and the predicted FD values (*b*_o_) and estimated age effects across each age subgroup are shown in Figures 3 and 4. Overall, the estimates of age effect were negative for all age subgroups until the age of 75 years, though stable (nonsignificant) during 5 years age span. The exception was for the 55–60 years age group in the left hemisphere (unadjusted *p* = .037) and for the 50–55 years age group in the right hemisphere (unadjusted *p* = .044) where age has a significant impact on complexity deterioration. Generally, for age subgroups, the mean estimated FD of the right and left hemispheres slightly increased until age 55 and 60 years then followed a u‐trend until age 75 years and then dropped afterward.

**TABLE 7:** Estimated effects of age (intercept [*b*_o_], estimate, standard error [SE], and significance *p*‐value) on each FD measure of each age subgroup

Age group (years)		L hemisphere	R hemisphere	L frontal	R frontal	L temporal	R temporal	L parietal	R parietal	L occipital	R occipital
45–50	*b*_o_	2.3838	2.3542	2.3960	2.3712	2.2695	2.2817	2.3339	2.3385	2.1908	2.1261
	Estimate	−0.0002	0.0002	−0.0014	−0.0006	0.0011	0.0004	−0.0010	−0.0008	0.0008	0.0024
	SE	0.001	0.001	0.001	0.001	0.001	0.001	0.001	0.001	0.001	0.001
	*p*‐Value	.713	.681	.097	.459	.189	.663	.237	.359	.504	**.047**
50–55	*b*_o_	2.3869	2.3876	2.3648	2.3741	2.3599	2.3287	2.3252	2.3344	2.24242	2.2649
	Estimate	−0.0002	−0.0004	−0.0006	−0.0007	−0.0007	−0.0007	−0.0005	−0.0007	−0.0002	−0.0004
	SE	0.000	0.000	0.000	0.000	0.000	0.000	0.000	0.000	0.001	0.000
	*p*‐Value	.239	**.044**	.056	**.021**	.059	**.043**	.160	.052	.713	.450
55–60	*b*_o_	2.3940	2.3769	2.3459	2.3669	2.3135	2.2941	2.2981	2.3213	2.2829	2.2656
	Estimate	−0.0004	−0.0002	−0.0004	−0.0006	0.0000	−0.0001	−0.0002	−0.0005	−0.0006	−0.0003
	SE	0.000	0.000	0.000	0.000	0.000	0.000	0.000	0.000	0.000	0.000
	*p*‐Value	**.037**	.320	.227	**.047**	.967	.813	.556	.108	.173	.462
60–65	*b*_o_	2.3863	2.3814	2.3490	2.3421	2.2963	2.2917	2.3014	2.3142	2.2425	2.2708
	Estimate	−0.0002	−0.0002	−0.0003	0.0000	0.0003	0.0000	−0.0001	−0.0003	−0.0002	−0.0003
	SE	0.000	0.000	0.000	0.000	0.000	0.000	0.000	0.000	0.000	0.000
	*p*‐Value	.195	.231	.265	.918	.278	.901	.638	.282	.693	.407
65–70	*b*_o_	2.3927	2.3889	2.3536	2.3520	2.3210	2.2857	2.3055	2.3355	2.2152	2.2367
	Estimate	−0.0003	−0.0003	−0.0005	−0.0003	−0.0001	0.0000	−0.0003	−0.0006	0.0003	0.0001
	SE	0.000	0.000	0.000	0.000	0.000	0.000	0.000	0.000	0.000	0.000
	*p*‐Value	.059	.073	.070	.166	.842	.993	.291	**.023**	.505	.846
70–75	*b*_o_	2.3999	2.4144	2.3280	2.3486	2.3131	2.2801	2.3080	2.3064	2.2744	2.2510
	Estimate	−0.0002	−0.0005	0.0001	−0.0003	0.0001	0.0002	−0.0001	−0.0003	−0.0005	−0.0004
	SE	0.000	0.000	0.000	0.000	0.000	0.000	0.000	0.000	0.001	0.001
	*p*‐Value	.292	**.019**	.857	.376	.694	.585	.809	.472	.301	.472
75–80	*b*_o_	2.3567	2.3797	2.2661	2.3295	2.3929	2.3831	2.4214	2.3317	2.1573	2.1560
	Estimate	0.0003	0.0000	0.0009	0.0001	−0.0012	−0.0012	−0.0018	−0.0005	0.0009	0.0007
	SE	0.001	0.001	0.001	0.002	0.002	0.002	0.002	0.001	0.002	0.002
	*p*‐Value	.739	.962	.511	.935	.450	.456	.256	.755	.664	.713

*Note*: Significant values are in bold.

Abbreviation: FD, fractal dimension.

At lobar level, a similar accelerated negative effect of age was present in the right frontal lobe for 50–55 years (unadjusted *p* = .021) and 55–60 years (unadjusted *p* = .047), right temporal lobe for 50–55 years age group (unadjusted *p* = .043), and right parietal lobe for 65–70‐years age group (*p* = .023), and positive effect of age in right occipital lobe for 45–50 year age group followed by a stable period (nonsignificant age effect) until the older age 75–80 years.

## 4 DISCUSSION

Using automated segmentation and parcellation, our results indicate that age‐related changes and sexual dimorphism in cerebral cortex complexity are not uniformly distributed across the brain. The right side of the brain was found to be the most complex while age‐related changes and sex differences in FD were significant in both hemispheres and all lobes except the left parietal lobe. While controlling for the effect of brain size, the estimated FD values were higher for females than males in the left hemisphere and left and right parietal lobe, and higher for males in the right temporal and left and right occipital lobe than for females. At the lobar level, the left parietal lobe showed the largest difference between the sexes whereas the right parietal lobe showed the largest effect of age on complexity. Our sex‐specific analysis showed significant age effects in both males and females, with relatively more age‐effected regions in male brains. The leftward hemispheric and temporal lobe asymmetry decreased with age and males had significantly lower asymmetric hemispheres and higher asymmetric parietal, and occipital lobes than females.

Our findings of higher FD values representing more complex structure in the right hemisphere, and regionally the right frontal lobe are according to our expectations (Lee et al., 2004; Liu et al., 2020). The normal brain ageing is associated with reduced cortical complexity (Krohn et al., 2019; Madan & Kensinger, 2016; Marzi et al., 2020), we also found a negative association of FD with advancing age in all regions. Our regional analysis for age differences agrees with the previous findings of FD studies (Jao et al., 2021) demonstrating that as adulthood progresses to old age, morphological alterations occur in all lobes of both hemispheres. We also found that the temporal lobe was least affected by age and frontal and parietal lobes were the most age‐affected regions as demonstrated earlier by Madan and Kensinger (2016). After AIC was applied, FD changes were best described by quadratic functions in the bilateral frontal lobe as reported by Liu et al. (2020) in a longitudinal study of 161 older adults (age ranged 70–90 years) at 2 and 6 years follow‐ups, indicating nonlinear complexity decline in frontal and right temporal lobe across adulthood, whereas the rest of all other regions showed linear change. The results from this study provide some additional information on brain shape‐related structural changes across adulthood by considering more refined unilateral parcellation of the cortex (i.e., lobes) which may be particularly useful when relating FD estimates to cognitive measures (Madan & Kensinger, 2016).

In the literature, there are limited studies on sex‐specific ageing of brain structures evaluated with FD analysis. Overall, FD decreased with ageing in both men and women (aged 38–80 years, M/F, 264/443), at the same location within the left insula, additional change in men was in the left subcentral gyrus while in women in the cingulate gyrus and left post central sulcus (Podgórski et al., 2021). In our study, FD of all structures showed a significant age‐related decline in males except the right temporal lobe where a trend of nonsignificant age effect was observed in both males and females (uncorrected *p*‐value .0445). On the other hand, the age effects were not significant in the female right hemisphere, left temporal, and left occipital lobes. Our findings of more pronounced and widespread effects of ageing in males agree with Jao et al. (2021), but are not in agreement with (Podgórski et al., 2021), reporting that cortical ageing is more complex in females. The relation between the cortical complexity decline and the clinical implication of its change is not known yet. However, comparable results have been reported in volumetric studies that during midlife, men exhibited greater brain reduction in mid‐line brain regions after correcting for the TBV loss (Guo et al., 2016). Similarly, the Baltimore longitudinal study of ageing has demonstrated a steeper rate of cognitive decline in men and greater resilience to age‐related cognitive decline in older women compared with men (McCarrey et al., 2016).

The results from this study provide an important refinement of our view of structural sexual dimorphism in brain cortical complexity. Earlier studies have reported either greater complexity of young female superior‐frontal and parietal lobes (Luders et al., 2004) or no sex differences in hemispheric lobes in older adults (Liu et al., 2020). In our study, without considering brain size adjustment, significantly higher FD values were found in the male brain in all regions (*p* < .001) except the left parietal lobe. While controlling for brain size, we found that females had significantly higher cortical complexity than males in the left hemisphere and left and right parietal lobe which agrees with earlier reports (Luders et al., 2004). Males showed significantly higher FD in the right temporal and left and right occipital lobes which are new findings. Luders et al. (2004) measured sex differences in a sample of age‐matched young adults (*n* = 60, mean age 24.3 ± 4.4 years). Moreover, the small‐sample studies on the male/female brain differences are particularly vulnerable to false‐positive and false‐negative findings because the distribution of sex‐related differences are mostly subtle and it is increasingly recognized that there is substantial overlap in the general population (Ritchie et al., 2018).

Increased cortical complexity implies more cortical surface area and folding area and has been shown to be significant in accounting for the FD of the cortical surface, with a positive coefficient in both hemispheres and several lobe regions (Im et al., 2006). Using the DK atlas in Freesurfer, Ritchie et al. (2018) studied sex differences in the surface area through sub‐regional analyses in the first release of UK Biobank MRI data (age range 44–77 years). They observed larger sex differences in the surface area where males had higher raw surface areas which were substantially reduced after adjustment of brain size as 18/68 larger in males and 9/68 larger in females indicating specific sex differences in these areas. Our findings of the reversed pattern of increasing cortical complexities in females might be explained as an adaptation to accommodate a larger surface area in a small volume (skull). The sexual dimorphism in cortical complexity might be associated with different cognitive functions and may thus provide possible explanation for functional and behavioural differences between males and females (Gur & Gur, 2017).

The regional changes in cortical complexity asymmetry are overlooked in the literature, yet an important aspect of healthy brain organization for many functions. As asymmetrically organized cortex leads to the increased proximity of collaborating brain regions and intrahemispheric clustering of specialized networks (Jacobs, 1999; Karolis et al., 2019; Ringo et al., 1994). We found rightward asymmetry of hemisphere, frontal, parietal, and occipital lobes and leftward asymmetry in the temporal lobe when assessed with paired sample *t* test. The rightward asymmetry of the hemisphere (Lee et al., 2004) and frontal lobe (Liu et al., 2020; Luders et al., 2004) are in line with previous reports but the rightward asymmetry of the parietal and occipital lobe opposes earlier reports (Liu et al., 2020; Luders et al., 2006). Our finding of leftward asymmetry in temporal lobe FD is not comparable with the above FD studies and is also a piece of novel information. However, our observed patterns of leftward and rightward asymmetries are consistent with the surface area asymmetries (Kong et al., 2018; Maingault et al., 2016), suggesting that asymmetry of cortical complexity may correspond anatomically to language and region‐specific functional lateralization, although further studies are needed to investigate both structure–function relationships in brain symmetry and ageing.

As we demonstrated, if brain size was included as a covariate to obtain age or sex‐specific effects on asymmetry, a positive effect, that is, increased leftward/decreased rightward asymmetry was observed only at the hemispheric level and no noticeable effect was seen at the lobar level. As suggested by Kong et al. (2018), increased brain size might lead to the development of additional sulci (Kang et al., 2015) which could have an impact on regional asymmetries as assessed with the Freesurfer atlas‐based approach (Desikan et al., 2006). In terms of age, the loss of hemisphere and temporal lobe asymmetries are in‐line with the surface area asymmetry loss (Kong et al., 2018), which has been reported for other surface based morphometric measure, that is, cortical thickness in a longitudinal adult lifespan sample indicating that asymmetry loss is a system‐wide process of ageing (Roe et al., 2021). As far as we are aware, no previous studies have reported possible age effects on the regional asymmetries of cortical complexity across adulthood except that by Lee et al. (2004) who reported that hemispheric asymmetry measured through FD on the skeletonized cerebral surface does not change with age or sex, by conducting two‐way ANOVA between young (*n* = 31, mean age 24.48 ± 5.02 years) and old (*n* = 31, mean age 63.23 ± 9.42 years) age groups. We found significant sex differences in asymmetry as females exhibited more leftward hemispheric asymmetry and males exhibited more rightward asymmetries of parietal and occipital lobe than did females and no sexual dimorphism in temporal lobe complexity asymmetries. These region‐specific structural asymmetries and their variation with age and between the sex groups not only contribute to the understanding of human brain asymmetry in the healthy population but also provide a reference source for future studies to explain the altered asymmetry in cognition and psychiatric disorders.

Regarding the trajectories of FD changes during 5 years age duration, we observed that overall FD remained stable in all regions though the age effect was negative and nonsignificant during this period. As demonstrated in the results section, accelerated age effects were noticed in right‐brain regions strengthening our previous finding of more vulnerability of the right brain for age effects at this narrow age range of 5 years across different stages of adulthood. Note that these findings are not corrected for multiple comparison corrections. The detection of such specific patterns highlights the complex degenerative process across adulthood and provides a platform that may contribute to a greater understanding of neuropathological features associated with degenerative disorders at different stages of ageing.

This study presents noteworthy strengths. First, the sample size is relatively large and was obtained from a single scanner, and population‐based setting, thereby increasing the generalizability of the results. Second, brain complexity is calculated using FD analysis which is more sensitive to age‐related cortical atrophy than volumes and other surface‐based measurements like cortical thickness and gyrification index (Lu, 2020; Madan & Kensinger, 2016; Madan & Kensinger, 2018). Third, it is suggested that the FD constitutes a useful measure of hemispheric asymmetry which is independent of brain size (Lee et al., 2004; Madan & Kensinger, 2016); however, we managed to quantify the age, sex, and regional cerebral asymmetries more completely than what was obtained from studies limited to asymmetry in sub shape characteristics such as length and volume while correcting for brain size. The sex differences in puberty and early adulthood may be particularly modulated by hormonal factors, in older adulthood environmental factors may have a greater impact. Therefore, future research should include these factors in the investigation of brain complexity to get a better picture of cognitive decline. A fundamental limitation to the interpretation of our results is the fact that age trends were estimated using cross‐sectional data. However, only a within‐subject longitudinal design allows the determination of true age‐related changes which would be possible to do in the future as UK Biobank is conducting the second phase of imaging for their participants. To date, only one longitudinal study (Liu et al., 2020) with 6 years age difference in older adults (70–90 years) has examined the age‐related complexity change in cortical lobes, and comparison with other markers of atrophy needs to be explored further in middle to older age adults. Since the UK Biobank consists of middle to older age adults, these findings cannot be generalized to younger <45 years or older results >79 years where linear modelling of cross‐sectional age effects across the different stages of adulthood and ageing samples is known to follow nonlinear brain trajectories over time. Other limitations include methodological differences in estimation of FD such as: (a) we computed FD on nonequally spaced points on a logarithmic scale, making results not comparable with most of recent works that, in contrast, calculated FD on equally spaced points on logarithmic scale (Goni et al., 2013; Krohn et al., 2019; Madan, 2018; Madan, 2019; Madan & Kensinger, 2016; Madan & Kensinger, 2017a; Madan & Kensinger, 2017b; Madan & Kensinger, 2018; Marzi et al., 2018; Marzi et al., 2020; Marzi et al., 2021; Pani et al., 2022; Pantoni et al., 2019) and (b) just for cerebral lobes, the fractal scaling window is smaller than one decade (0.7), unlike mathematically generated fractals, real data cannot be ideally fractal over all scales and the range of scaling window is still under debate (Marzi et al., 2020; Meregalli et al., 2022), it is suggested to be at least over one decade (Caserta et al., 1995) or at least two orders of magnitude (Losa, 2009); however, relatively small scaling range, mainly between 0.5 and 2 order of magnitude is also observed in neuroscience (Di Ieva, 2016).

## 5 CONCLUSION

Our large‐scale complexity study offers an excellent starting point for future research on the role of sex‐specific brain anatomy for cognition, emotional and behavioural differences between males and females. Moreover, our findings may help to explain the sex differences in diagnosis and prognosis of the number of neurological disorders.

## FUNDING INFORMATION

This research has been conducted using the UK Biobank resource under application number 24089 (PI Waiter). This work was supported by the Aberdeen Biomedical Imaging Centre with financial support from the Roland Sutton Academic Trust (RSAT‐0067/R/19). UK Biobank received ethical approval from the North West Multi‐Centre Research Ethics Committee (11/NW/03820). All UK Biobank methods were performed following the UK regulations. All UK Biobank participants gave written informed consent.

## Data Availability

The data sets processed and analysed during the current study are available from the online open access UK Biobank repository (https://www.ukbiobank.ac.uk/). This research was conducted under the UK Biobank Resource under Application Number 24089 (PI Waiter).
